# Pilot study to test the Slovenian version of the Kennerley–Gath Blues questionnaire

**DOI:** 10.18332/ejm/142107

**Published:** 2021-10-18

**Authors:** Mirjam Golež, Polona A. Mivšek

**Affiliations:** 1 Midwifery Department, Faculty of Health Sciences, University of Ljubljana, Ljubljana, Slovenia; 2 Celje Maternity Hospital, Celje, Slovenia

**Keywords:** screening, postnatal well-being, Blues Questionnaire, midwife support, validation of the tool

## Abstract

**INTRODUCTION:**

Postpartum blues in 20% of cases develops into postnatal depression if it lasts longer than 14 days, so the condition requires attention. To help Slovenian midwives in screening for postpartum blues, we aimed to translate the Kennerly–Garth Blues Questionnaire (BQ).

**METHODS:**

The blues questionnaire was translated using a double-blind translation method. The Cathedra for Midwifery at the Faculty Health Sciences Ljubljana reviewed the ethics and research design of the study. The online survey was conducted among Slovenian postpartum women who had to be between the 3rd and 15th day postpartum (inclusion criteria). A snowball sampling was used. The online questionnaire was active from January to March 2020. Women voluntarily participated in the survey and were assured of anonymity.

**RESULTS:**

A total of 101 women participated in the study. More than half (58%) scored ≥7 points in the questionnaire, which is the cut-off score, indicating postpartum blues. More single women obtained a high score (66.6%) than those who were married (63.6%) or in an extramarital relationship (50.9%). High questionnaire scores were more common among women who had had their second child. Cronbach alpha for the Slovenian version of the Blues questionnaire was 0.995.

**CONCLUSIONS:**

The survey instrument can be used easily and quickly and is a good way to open discussion with women about emotional and mental health in the postnatal period. The Slovenian version of the Blues questionnaire showed a satisfactory level of internal consistency, but a larger study should be conducted to evaluate the cut-off score and the content validity.

## INTRODUCTION

Researchers report varying prevalence of postpartum blues; a recent population-based study estimated a prevalence as high as 85%^1^. The majority of authors blame altered hormonal balance due to the absence of the placenta as the main reason for postpartum emotional disturbance of women^2,3^, but some caution that psychosocial issues must also be considered as a contributing aetiology^1,4^. Postpartum blues do not require treatment, but women do need support and understanding and, most importantly, an explanation of what is going on. The problem usually resolves itself within a few hours or days, but persistent cases can lead to postnatal depression in 20%^2,5^. Postnatal depression is a much more serious condition as it also affects the baby, it impacts mother–child interaction and consequently child development. It would therefore be useful to screen women for postpartum blues. The Kennerley– Gath Blues Questionnaire (BQ) is a screening instrument for postpartum blues. To the authors’ knowledge, this is the only specific screening questionnaire for postpartum blues. It is composed of 7 groups (clusters) of signs of postpartum blues. Women rate whether their feelings are different from usual and their answers are scored with a maximum of 28 points. A woman is at risk for postpartum blues if she collects ≥7 points^6^. The aim of the study was to translate the BQ into Slovenian and to conduct a pilot study to validate the instrument for internal consistency.

## METHODS

A non-experimental method of empirical research was used, supported by an online survey as a data collection technique. The Kennerley–Garth Blues questionnaire was used as it is specific to postpartum blues and has been validated by the authors (the survey showed good content validity and 7 clusters were found to be robust enough to measure blues). The research instrument was translated through a double-blind translation process; first from English to Slovenian (by the first translator) and back from Slovenian to English (second translator). The original English version and the translated English version were compared and differences were addressed within the research team (consisting of midwives and professors of English).

The research design with the ethical issues of the study was approved by the Cathedra of midwifery in the Faculty of Health Sciences that is responsible for the profession development and research.

The questionnaire was posted on social networks where postpartum women engage and a snowballing method was used. Participation in the study was anonymous and voluntary. Inclusion criteria were: gender (female), and motherhood (delivered 3–15 days ago). The survey was active from 16 January to 19 March 2020. We used the 1KA online survey tool (https://www.1ka.si/) to gather and analyze the data. Basic descriptive statistics were performed on the data. For validation of the tool, we calculated the Cronbach α as an internal reliability measure.

## RESULTS

A total of 101 women met the inclusion criteria for participation in the study (gave birth 3–15 days ago). For half of the participants (n=51; 50.5%), this was their first child; the others (n=50; 49.5%) already had children. Of the participants, 51 women lived with a partner but were not married (50.5%), 44 were married (43.6%), and 6 (5.9%) were single (divorced, widowed, or single).

The most common feelings of postnatal period that were reported by participants were: tiredness (83%), feeling happy (80%), brooding about things (74%), and changeable mood (73%). Table 1 summarizes participants’ responses about how they felt during the postpartum period compared to how they usually feel; 28 feelings are listed in the BQ. Answers ‘less than usual’ and ‘much less than usual’ were combined into ‘less than usual’; answers ‘more than usual’ and ‘much more than usual’ were combined into ‘more than usual’. Women reported being ‘more than usual’ tired (85%), happy (67%), prone to be broody (66%), and tearful (64%). They also reported being able to concentrate ‘less than usual’ (47%), wanting to be alone ‘less than usual’ (45%), and being less numb ‘less than usual’ (42%). The Cronbach alfa for the 28 statements was 0.995.

**Table 1:** Proportion of participants reporting differences in feelings postnatally (N=101)

*Feelings*	*Less than usual n (%)*	*As usual n (%)*	*More than usual n (%)*
Tearful	10 (10)	27 (27)	64 (64)
Mentally tense	16 (16)	38 (38)	47 (47)
Able to concentrate	47 (47)	42 (42)	12 (12)
Low spirited	32 (32)	44 (44)	25 (25)
Elated	20 (20)	30 (30)	51 (51)
Helpless	17 (17)	48 (48)	36 (36)
Find it difficult to show feelings	15 (15)	58 (57)	28 (28)
Alert	18 (18)	38 (38)	45 (45)
Forgetful, muddled	8 (8)	33 (33)	60 (60)
Anxious	16 (16)	59 (58)	26 (26)
Wish to be alone	45 (45)	43 (43)	13 (13)
Mentally relaxed	33 (33)	49 (49)	19 (19)
Brooding on things	10 (10)	25 (25)	66 (66)
Feeling sorry for myself	29 (29)	55 (54)	17 (17)
Emotionally numb	42 (42)	52 (51)	7 (7)
Depressed	35 (35)	49 (49)	17 (17)
Over-emotional	10 (10)	28 (28)	64 (64)
Happy	17 (17)	17 (17)	67 (67)
Confident	28 (28)	46 (46)	28 (28)
Changeable spirit	8 (8)	38 (38)	55 (55)
Tired	2 (2)	14 (14)	85 (85)
Irritable	11 (11)	49 (49)	50 (50)
Crying, not able to stop	25 (25)	54 (53)	22 (22)
Lively	30 (30)	42 (42)	29 (29)
Over-sensitive	12 (12)	35 (35)	54 (54)
Up and down in the mood	9 (9)	55 (54)	37 (37)
Restless	19 (19)	47 (47)	35 (35)
Calm, tranquil	31 (31)	43 (43)	27 (27)

We calculated that 59 (58%) of the women who participated in the study collected ≥7 points on the BQ. Postpartum blues was more common in women who were having their second child (63%) compared to women who were giving birth for the first time (55%) or had more than one child at home (58%). Women who were single were more likely to have postpartum blues (66.6%) compared to women who were married (63.6%) or living with their partner in an extramarital relationship (50.9%).

## DISCUSSION

Researchers estimate postpartum blues to be common; different studies show varying rates of women experiencing postpartum blues^1,4^. Results may be influenced by the research instrument used to determine postpartum blues. Ntaouti et al.^1^, who used the BQ, found that 43.1% of postpartum women collected ≥7 points (and were thus defined as having postpartum blues). In our study, the percentage was slightly higher at 58.4%. This could be because Ntaouti et al.^1^ surveyed their participants on the 3rd postnatal day in hospital (before discharge). In order to recruit at least 100 participants, we defined broader inclusion criteria (women from day 3 to day 15 postpartum could participate in the study), similarly to an Italian study that used BQ^7^. Some women in our study were already at home when they completed the questionnaire (in Slovenia, women are discharged from the maternity hospital on day 3 postpartum). Some women might feel more insecure, be more anxious and afraid of taking care of the baby alone at home without medical professionals nearby to advise and support them, and this might be the reason why we had a higher proportion of women experiencing postpartum blues. The second reason for the difference in prevalence could be that Ntaouti et al.^1^ excluded women with depressive symptoms (those who scored 13 or more on the EPDS) before performing the BQ, whereas we did not.

Ntaouti et al.^1^ found that primiparas are more prone to postpartum blues than multiparas. Our study showed opposite results; more women with a second baby scored higher on BQ than primiparas. The reason could be that caring for a newborn and a toddler can be very demanding and stressful. Future studies should take into account the age of the siblings and other factors that might affect the postnatal well-being of the women (e.g. long tiring births, intrapartum complications, etc.). The women with a third child had lower scores in BQ than the women with a second child, which could be because they already had experience in caring for a newborn and older child at the same time and had more realistic expectations of life with multiple children in the postpartum period. Maliszewska et al.^4^ examined sociodemographic factors that might influence postpartum blues. One of their findings was that partnership may protect women from the blues. Our results confirmed this (women in partnerships scored lower on the BQ than single women), however, our sample of women without partners was very small and the differences between groups were not large, so the results must be interpreted with caution. Since partnership quality is also an important factor^4^, further studies should also consider partnership satisfaction when examining postpartum mental health.

Grussu and Qutraro^7^ claim that postpartum blues might also be associated with postpartum anxiety. First-time mothers in particular might feel insecure and therefore the feeling of overall responsibility for the newborn might be overwhelming for them. Grussu and Qutraro^7^ observed postpartum women 15 days after delivery. They found that women had more anxiety two days after discharge from the hospital; these were the days when women were most anxious. Ntaouti et al.^1^ also found similar apprehension, but they did not observe the women after they left the maternity hospital. Based on these results, we expected that more women in the group of participants who completed the BQ at 3–7 days postpartum would score higher than those in the 8–15 days postnatal group. Surprisingly, more women who were 8–15 days postpartum scored higher. The reasons for this could be many. One could be the lack of support; usually this is the time when the partner returns to work (Slovenian men usually take a week of paternity leave).

The average time to complete the questionnaire by women was less than one minute. It took the researchers less than three minutes to calculate the scores manually. If we were to create an online questionnaire where the scores are calculated automatically, this could save midwives and nurses time in the postpartum period and make screening easier. However, we must remember that the conversation about postnatal mental health is most important; the screening tool cannot replace the communication between a midwife and a woman. With the conversation we could define what support a woman needs (emotional, informational or any other kind) or whether she just needs to be reassured that her feelings are normal.

The reliability of the Slovenian version of the tool, assessed by the Cronbach alpha test, was sufficient but further validation of the Slovenian version of the BQ should be performed (factor analysis should be used to redefine the suitability of clusters and cut-off score should be checked for the Slovenian population). The sample in our study does not provide the possibility to generalize the results, but it can provide useful insights.

We have used the BQ which, to the authors’ knowledge, is the only screening instrument specifically for postpartum blues. There have been some efforts in the past to develop a scale for screening postpartum blues^1^, but the instruments have never been validated.

### Strengths and limitations

Our study has certain limitations. Despite the awareness of risk in using the internet for a survey, we conducted an online survey because we assumed it would be easily accessible to participants on a mobile phone and therefore the response rate would be better in comparison to conducting the research with paper questionnaires. Because the study was conducted during the COVID-19 epidemic, the online survey was the only way to reach postpartum women. We think the response rate was high considering that women have a lot on their plate in the first few days postpartum, but it could also be that participants who felt emotionally distressed were more motivated to participate in the study than others (and therefore we have a slightly higher proportion of women with blues than other researchers)^1^.

## CONCLUSIONS

The BQ could be a suitable tool for screening for postpartum blues. It is simple and quick to perform and interpret, but most importantly its value lies in opportunity to initiate conversation with the woman about her mental state. Despite the fact that postpartum blues is a mild mental disorder that does not need to be cured, women would benefit from additional support and attention from the midwife. In our study, we confirmed good internal reliability of the Slovenian version of the BQ, however, further studies should be conducted to confirm the cut-off score of the Slovenian version of the instrument and to re-evaluate the content of the 7 main clusters of the questionnaire.

## Data Availability

The data supporting this research are available from the authors on reasonable request.
